# Malignant ameloblastoma (metastatic ameloblastoma) in the lung: 3 cases of misdiagnosis as primary lung tumor with a unique growth pattern

**DOI:** 10.1186/s13000-015-0367-0

**Published:** 2015-07-25

**Authors:** Rui Bi, Lei Shen, Xiongzeng Zhu, Xiaoli Xu

**Affiliations:** Department of Pathology, Fudan University Shanghai Cancer Center, 270 Dong An Road, Shanghai, 200032 China; Department of Oncology, Shanghai Medical College, Fudan University, 270 Dong An Road, Shanghai, 200032 China

**Keywords:** Malignant ameloblastoma, Lung, Metastatic, Differential diagnosis

## Abstract

Malignant ameloblastoma (metastatic ameloblastoma, MA) is currently defined as a distinct pathologic entity, MA, despite its histologically benign appearance. According to the new criteria, the histological and clinical features of MA are more homogenous. Here, we report three cases of histologically confirmed pulmonary MA. Two of the three patients complained of chest pain as the primary symptom, and the other case was detected upon the evaluation of pulmonary nodules found during a health examination after a local recurrence of mandible ameloblastoma. All three patients were female with an average age of 48 years. The intervals between the primary ameloblastoma and metastasis to the lung were 14 years, 19 years and 10 years, averaging 14.3 years. Prior to metastasis to the lung, only one patient experienced local recurrences, at 5 and 19 years after the primary tumor resection, while the other two patients both remained disease-free. Computed tomography (CT) or X-ray evaluation demonstrated multiple bilateral lung nodules ranging in size from several millimeters up to 2 cm. Histologically, the pulmonary metastatic tumors showed a unique growth pattern: the tumor cells grew among the interstitial alveoli but did not appear to destructively infiltrate the surrounding tissue. Immunohistochemically, the MA cells expressed squamous differentiation markers, such as CK10/13 and p63, while the alveolar epithelial cells stained for TTF1 and PE10. In this paper, we discuss the clinical behavior, differential diagnosis and unique growth pattern of pulmonary MA.

## Background

Ameloblastoma is a rare odontogenic epithelial tumor that represents only approximately 1 % of all jaw tumors, but it is the second-most common odontogenic tumor. Although it is always considered a benign odontogenic tumor, ameloblastoma is slow growing, locally aggressive, and has a high propensity for local recurrence if not removed completely. Some authors tend to regard it as a potentially malignant tumor [1, 2], but metastasis is rare. However, a histologically benign-appearing ameloblastoma can metastasize to local lymph nodes or other distant organs, such as the brain, lung, skin, etc. Over a decade can pass before metastatic tumors are observed after the resection of the primary tumor [3, 4]. Histologic appearance alone cannot indicate late metastasis. Because of its complex behavior, ameloblastoma continues to be a subject of intense interest and some controversy.

The WHO classification of odontogenous tumors (2005) currently defines malignant ameloblastoma (MA) as “an ameloblastoma that metastasizes in spite of a benign histological appearance.” Ameloblastoma with cytological atypia is defined as ameloblastic carcinoma even if metastasis is absent. Thus, MA is defined as a retrospective diagnosis that can only be made when metastasis occurs. In many cases, MA not only maintains the histological characteristics of the parent tumor but also continues to display similarly indolent clinical behavior. However, the histological features of MA of the lung are seldom discussed in the literature. Because of the low frequency of MA and its unclear clinical history, physicians should avoid misdiagnosing MA as other primary or metastatic tumors of the lung.

## Case Presentation

### Patients and samples

This study included 3 patients from the Department of Pathology, Fudan University Shanghai Cancer Center, who were diagnosed between 2010 and 2014. Patient 1 was an inpatient of our hospital, and patients 2 and 3 were accepted for consultation. The clinical information and gross features were collected from the referring hospitals and the case files of our hospital. Formalin-fixed paraffin-embedded tissue blocks or unstained slides for the consultation cases were reprocessed for hematoxylin-eosin staining and immunohistochemistry. Slides of one of the local recurrences of patient 2’s mandible tumor were also available. Follow-up information was available in all three cases.

### Immunohistochemistry

Immunohistochemistry was performed on three pulmonary MAs and one mandible ameloblastoma. Cytokeratin (dilution 1:150, Dako, clone AE1/AE3), EMA (dilution 1:100, Dako, clone E29), CK7 (dilution 1:200, Dako, clone OV-TL12/30), p63 (dilution 1:50, Dako, clone 4A4), TTF-1 (dilution 1:100, Leica, clone SPT24), SP-A (dilution 1:50, LongIsland, PE10), CK5/6 (dilution 1:200, Dako, D5/16 B4), CK10/13 (dilution 1:100, Dako, DE-K13), and vimentin (dilution 1:1000, clone V9, Dako) were all used a Ventana Benchmark XT autostainer (Ventana Medical Systems Inc., Tucson, AZ, USA). Appropriate positive and negative controls were included.

## Results

### Clinicopathological data (Table 1)

**Table 1:** Clinicopathologic Features of 3 metastastic lung tumor of the ameloblastoma

Case No	Age (year)	Clinical manifestation	Imaging manifestation	Side of the lung tumor	Tumor size of the lung tumor	Intervals between the primary ameloblastoma and matastasis to lung	Local recurrence in oral	Follow-up
1	52	mild chest pain for one month	multiple nodules	bilateral	A few mm to 2 cm	14 years	no	57 months
2	48	routine examination after ameloblastoma local recurrence	multiple nodules	bilateral, mainly in the right lung	A few mm to 2 cm	19 years	The first and sencond local recurrence was 5 and 19 years after the oral surgery, respectively	53 months
3	44	mild chest pain for 10 days	multiple nodules	bilateral	A few mm to 1.8 cm	10 years	no	9 months

mm: millimeters; cm: centimeters

**Fig. 1:**
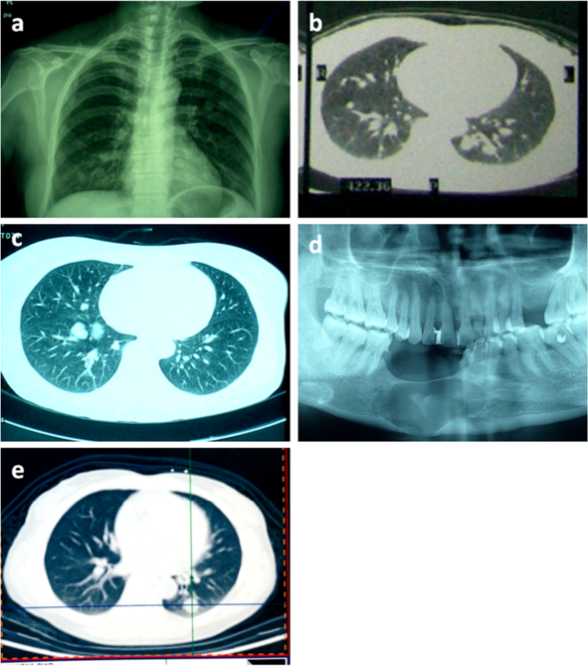
X-ray and CT findings of MA. (**a**) and (**b**): X-ray and CT showing bilateral pulmonary nodules in patient 1. (**c**): CT scan showing bilateral pulmonary nodules that are mainly located in the right lung in patient 2. (**d**): X-ray of recurrent ameloblastoma in patient 2 showing a cystic radiolucency with scalloped and hyperostotic borders below the former surgical region involving the inferior border of mandible. Expansile destruction of the inferior mandibular cortex was seen. (**e**): CT scan showing bilateral pulmonary nodules in patient 3. The lobular or irregular round nodules with invariable sizes in all the three cases

The three female patients were 52, 48 and 44 years old (mean, 48 years old) at the time of the confirmation of the metastatic lung tumor. Two patients experienced chest pain as the primary symptom (case 1 and 3) and did not have a history of odonto-tumors. Patient 2 had undergone routine examinations due to the local recurrences after the original surgery for ameloblastoma 19 years ago. We investigated the clinical histories of the other two patients and determined that they had undergone ameloblastoma surgery approximately 14 years and 10 years before. All primary tumors were located in the mandible and metastasized to the lung after 10-19 years (mean, 14.3 years). X-ray and CT imaging showed multiple nodules in the bilateral lungs that measured several mm up to 2 cm in three cases (Fig. 1) and were mainly located in the right lung in patient 2. They demonstrated some lobular or irregular shape nodules with invariable sizes in radiology. X-ray of recurrent ameloblastoma in patient 2 showed a cystic radiolucency with scalloped and hyperostotic borders, which was below the former surgical region involving the inferior border of mandible with expansile destruction of the inferior mandibular cortex (Fig. 1). Positron emission tomography (PET)-CT showed that the fluorodeoxyglucose (F18-FDG) metabolism of the nodules was normal in patients 1 and 3. Tumor markers in serum for lung cancer, including SCC, NSE, CA125, CEA, Cyfra21-1 were normal in all the three cases. One nodule in each patient was removed with a thoracoscopy for biopsy. No subsequent therapy was administered.

**Fig. 2:**
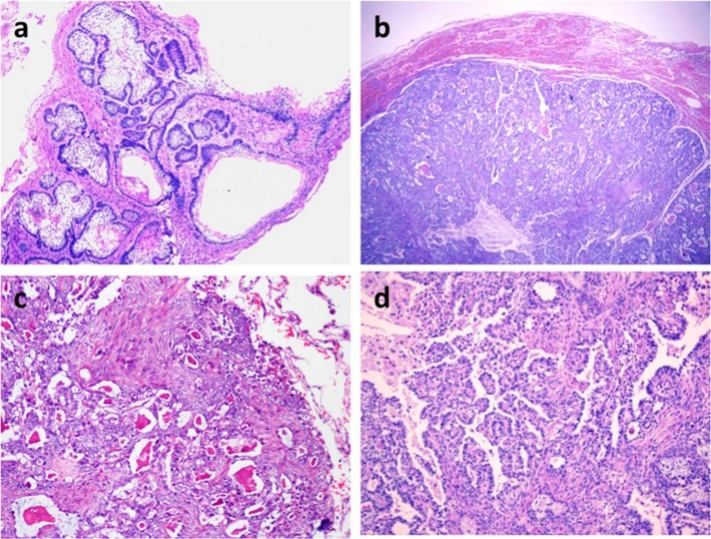
Histological findings of MA. (**a**): The locally recurrent tumor showed a solid/multicystic-type ameloblastoma. The tumor nests consisted of stellate cells with peripheral palisading. The stroma was fibrous. HE × 40. (Case 2). (**b**): The pulmonary metastatic lesion was more cellular, with a clear margin between the lesion and the surrounding lung tissue. HE × 40. (**c**): Spindle cells and oval cells comprised the nests and intercrossed the margin with glandular structures. (**d**): Many glandular/papillary structures were observed, among which were sheets of spindle cells with local squamous metaplasia. Cytological atypia was absent. HE × 100

**Fig. 3:**
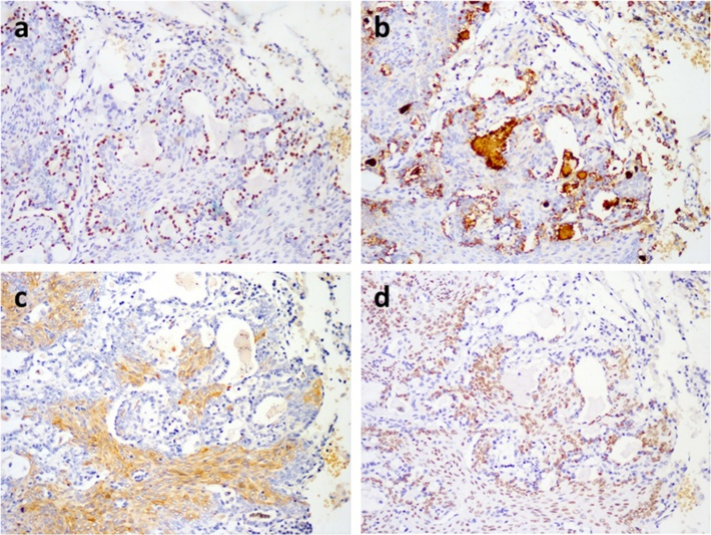
Immunohistochemical staining in MA. (**a**) and (**b**): The lining epithelial cells of the gadular structures are TTF-1+ and PE10+, indicating the residual alveolar epithelial cells (×100). (**c**) Stellate cells are CK10/13+ (×100). (**d**) The spindle cells are also p63+ (×100)

The two consultation cases were initially not clearly diagnosed and were thought to be benign lesions because of the unremarkable morphology (cases 2 and 3). The morphological description for patient 1 was based solely on the frozen section diagnosis.

Patient 1 was biopsied at our hospital. Grossly, the nodule was relatively soft and well circumscribed from the surrounding lung tissue. The cut surface was solid, grey and white, without obvious cysts or necrosis.

Histologically, the pulmonary nodules of the three patients were all well circumscribed and interspersed by many glandular/papillary structures and cellular cords or nests (Fig. 2a and Fig. 2b). The glandular/papillary elements consisted of columnar or cuboidal epithelial cells with some serous secretions in the lumens. The cellular nests among the glandular/papillary structures contained mostly spindle or ovoid tumor cells with a poorly defined cytoplasm (Fig. 2c). Focal squamous metaplasia could be observed (Fig. 2d). Cytological atypia, mitosis and necrosis were absent. However, the mesenchymal elements of the primary “ameloblastic fibroma” according to the original diagnosis were absent from the metastatic tumor in patient 1. In patient 2, the locally recurrent lesion of the left mandible demonstrated the classic morphological features of a solid/multicystic ameloblastoma. The ameloblastoma nests consisted of stellate cells with peripheral palisading that mainly represented a follicular growth pattern. The nests were separated by a fibroblastic stroma. Many tumor nests demonstrated central squamous metaplasia and keratin pearl formation, which is characteristic of acanthomatous ameloblastoma. The infiltration of the local bone was observed. Compared with the local recurrent tumor, the pulmonary metastatic tumor appeared more cellular. In patient 3, further pathological details of the primary ‘ameloblastoma’ could not be obtained. In addition to the morphology described above, larger sheets of ameloblastoma cells with small squamous epithelium-covered cysts were observed in the pulmonary tumor.

### Immunohistochemistry

Immunohistochemical staining showed that the glandular/papillary elements were AE1/AE3+, EMA+, CK7+, TTF-1+ (Fig. 3a), PE10+ (Fig. 3b), p63-, CK5/6-, CK10/13-, and vimentin-, which demonstrated that they were alveolar epithelial cells. Moreover, the nested spindle/ovoid tumor cells among the alveoli were AE1/AE3+, EMA+, CK7-, TTF-1-, PE10-, p63+ (Fig. 3d), CK5/6+, CK10/13+ (Fig. 3c), and vimentin-. These immuohistochemical staining results indicated that the nested spindle/ovoid cells were ameloblastoma elements. The total Ki-67 index was approximately 1 %.

### Follow-up

None of the patients received further treatment from the time of the lung biopsies to the latest follow-up (March 2015). All three patients presented an eventless course. The lengths of the follow-up intervals were 57 months, 53 months and 9 months (mean, 39.7 months).

## Discussion

Previous studies have reported MA as “malignant”, “metastatic”, “metastasizing”, “ameloblastoma with metastasis”, etc. and have lacked uniform diagnostic criteria. Metastasis occurring from “adamantinoma of the jaw” was also included in this group. MA has been reported as a heterogeneous clinico-pathological entity that consists of ameloblastomas with different histological and clinical behaviors—from very aggressive to highly indolent. Because cytological atypia are not excluded from the early diagnostic criteria for MA, many reported cases of MA may be malignant entities other than MA [5, 6]. More than 100 cases of so-called MA have been reported since the first report in 1923 by Emura [7]. The valid identification of MA remains a problem.

However, the current WHO classification of odontogenic carcinomas clearly distinguishes between MA and ameloblastomatic carcinoma using the typical well-differentiated cellular features of MA [8]. According to the new definition, MA is less documented in literature. By reviewing 98 cases of MA reported in the literature, Van Dam et al. identified only 24 valid cases of MA based on the printed histological pictures and identified 3 additional cases [1, 6]. They found that MA can be isolated to a more homogenous clinical pathological entity with special clinical behavior based on histological criteria. They proposed that if aggressive clinic behavior is observed, such as rapid growth and widespread metastasis, ameloblastic carcinoma or other carcinomas should be suspected. We reported another 3 cases of pulmonary MA that were diagnosed using the WHO criteria, similarly to Van Dam.

MA most frequently metastasizes to the lung at a rate of approximately 75-88 % of metastases, and this rate does not differ by gender [1, 6, 7]. Approximately 80 % of the primary sites of pulmonary MA are located in the mandible, and the remaining cases are maxillary. Pulmonary MA commonly develops via hematogenous and lymphatic routes. In addition, a few metastases are assumed to have occurred due to aspiration, because the tumor grew within the bronchi and bronchioli. This assumption is further sustained because such tumors are often located in the right lung [9]. The disease-free interval of MA is usually quite long—ranging from 2 months to 45 years (mean, 14-18 years) [4, 3]. Interestingly, despite many local recurrences and widespread metastatic tumors, many pulmonary MA cases show indolent clinical behavior with long survival times. Longevity is also possible [10, 11]. Moreover, patients have died of causes other than MA metastasis in some vital cases [12]. Surgical excision is the only treatment option, because both radiotherapy and chemotherapy are ineffective. In cases of multiple pulmonary metastases, such as our patients, a wider excision has not been shown to be helpful.

The histological features of pulmonary MA have been insufficiently discussed in the literature. Henderson et al. noted that a metastasizing tumor in the lung was more cellular than the primary tumor [13]. Their histological pictures showed many glandular structures. The same morphology was also noted in a micrograph provided by Mantin O et al. [14]. Chou YH et al. indicated an interesting interstitial growth pattern of the MA cells in the lung [15]. We also observed this distinctive finding in all 3 of our cases. In case 2, the locally recurrent mandibular tumor was a solid/multicystic ameloblastoma with a follicular growth pattern. The nests consisted of a stellate reticulum with peripheral palisading basal cells and local squamous metaplasia separated by fibrous stroma. The pulmonary metastatic tumor of patient 2 was more cellular, with many glandular/papillary structures that did not appear in the primary tumor. Among these structures, nests of spindle/ovoid cells with peripheral palisading basal cells and local squamous metaplasia that resembled acanthomatous ameloblastoma were observed. The primary tumors of patients 1 and 3 were not available, but the metastatic tumors also presented similar histological features. The immunochemical staining results identified the ameloblastoma elements among the alveoli of the lung.

In our series, these cases were all initially thought to either clinically or pathologically represent a primary lung tumor, especially the two patients without a clear clinical history. After biopsy, the biphasic morphology initially confounded the preliminary diagnosis. The differential diagnosis included sclerosing hemangioma, adenoma and metastatic lesions, such as adenocarcinoma. We immunohistochemically stained one locally recurrent (patient 2) and 3 metastatic tumors in all three cases. In all tumors, the epithelial components were positive for cytokeratins. Particularly, the nests of spindle/ovoid cells in both the primary and metastatic tumors were CK5/6+, CK10/13+ and p63+, which indicated squamous differentiation. Only the pulmonary metastastic lesions were CK7+, TTF1+ and PE10+, which highlighted the glandular/papillary structures composed of hyperplastic alveolar epithelial cells that may have been stimulated by the non-destructive growth of the ameloblastoma. This non-destructive pattern may at least in part explain the indolent clinical behavior of pulmonary MA, because the metastatic tumor exists in the lung as an interstitial growth for a long time. However, more case observations are needed to support this hypothesis. Furthermore, the two types of hyperplastic cells and this unique growth pattern are important clues for the diagnosis of pulmonary MA.

Because of the limited collection of valid MA cases, the definite histopathological and clinical features of pulmonary MA remain to be clarified. Will metastatic tumors develop different histological patterns? If dedifferentiation occurs in a metastatic tumor [2, 16], should it still be identified as MA? To our knowledge, certain benign tumors, such as leiomyoma, can also metastasize to the lung [17]. Further investigations that focus both on the histopathology and clinical behavior as well as the molecular mechanisms of MA are mandatory to elucidate the mysterious nature of this tumor.

## Conclusion

MA is too rare to estimate its prognosis. Based on our results and the cited works, MA presents an indolent clinical behavior that is often identified more than 10 years after the resection of the primary ameloblastoma. In our experience, more active treatment approaches, such as excision or chemo- or radiotherapy are not necessary. The key point to the successful treatment of MA is its precise diagnosis in order to distinguish it from other primary or metastatic pulmonary tumors, especially when the clinical history is not clear.

## Consent

The data were obtained from the consultation and archives of the department. A copy of the written consent is available for review by the Editor-in Chief of this Journal.
